# CHRONIC KIDNEY DISEASE, MUSCLE WEAKNESS, AND MOBILITY LIMITATION

**DOI:** 10.1093/geroni/igz038.1926

**Published:** 2019-11-08

**Authors:** Kenzie Latham-Mintus, Simit Doshi, Ranjani Moorthi

**Affiliations:** 1 IUPUI, Indianapolis, Indiana, United States; 2 Indiana University, Indianapolis, Indiana, United States; 3 Indiana University Purdue University Indianapolis, Indianapolis, Indiana, United States

## Abstract

Objectives: Chronic kidney disease (CKD) is associated with increased mobility limitation. Prior research has documented that peripheral nerve abnormalities occur early in CKD and progressively worsen. Loss of balance, impaired muscle strength, and slow gait predispose older adults to falls and frailty. However, the current literature is limited by a lack of nationally representative data that includes objective measures of kidney disease and physical functioning. Thus, this research examines whether CKD is associated with muscle strength, balance, gait, and self-reported mobility limitations. Methods: Data come from the 2016 Health and Retirement Study (HRS). Estimated GFR, a measure of kidney functioning derived from creatinine levels in the blood, was used to classify CKD (i.e, eGFR<45 or Stage 3b CKD). Logistic and linear regression models were generated to examine the association of CKD with physical functioning, net of demographic characteristics (i.e., age, sex, race, and education) and comorbidities (i.e., obesity, pain, and number of diagnosed medical conditions). Results: In unadjusted models, CKD was significantly associated (p<0.05) with more mobility limitations, slower walking speeds, stronger grip strengths, and non-participation in balance tests. After adjusting for covariates, CKD (β=-1.43, p=0.01) was negatively associated with grip strength. In sex-stratified models, CKD was associated with slower walking speeds among men, whereas CKD was associated with more mobility limitations among women. Discussion: In a nationally representative sample of older adults, CKD was associated with poorer physical functioning on multiple measures. After adjusting for demographic characteristics and comorbidities, CKD was associated with increased muscle weakness.

